# Supplementary material for: Studying the genetics of participation using footprints left on the ascertained genotypes
Source: Nat Genet. 2023 Jul 13;55(8):1413–20. doi: 10.1038/s41588-023-01439-2 (PMC10412458; doi:10.1038/s41588-023-01439-2)
Supplement: Supplementary file 1 — Supplementary Tables 1–3 and Note. [file 41588_2023_1439_MOESM1_ESM.pdf]

nature genetics

Technical Report https://doi.org/10.1038/s41588-023-01439-2

Studying the genetics of participation using footprints left on the ascertained genotypes

In the format provided by the authors and unedited

Table of Contents

Supplementary Tables **2**

Supplementary Table 1: Unadjusted TNTC and WSPC PGS associations with phenotypes. **2**

Supplementary Table 2: LD score regression estimates of genetic correlations between primary participation and traits using WSPC results. **3**

Supplementary Table 3. Heritability estimates for liability scores underlying participation traits. **4**

Supplementary Note **5**

1. Identifying relatives **5**

2. *t*-statistics, standard errors, and sample sizes **5**

3. The major allele bias **7**

4. Additional quality filtering of phased SNPs **10**

5. Phenotypes **11**

5.1 Educational attainment 12
5.2 Age at first birth (AFB) 13
5. 3 Body mass index (BMI) 13
5.4 High-Density-Lipoprotein (HDL) cholesterol 13
5.5 Height 14
5.6 Glycated haemoglobin (HbA1c) 14
5.7 Number of siblings (N. siblings) 14
5.8 Number of children (N. children) 15
5.9 Glucose 15
5.10 Vitamin D 15
5.11 Sex hormone binding globulin (SHBG) 16
5.12 Grip strength 16
5.13 Lipoprotein A 17
5.14 Testosterone 17
5.15 Dietary study invitation 17
5.16 Dietary study participation 18
5.17 Physical activity study invitation 18
5.18 Physical activity study participation 18

6. Genetic components captured by TNTC, WSPC and BSPC **18**

7. Adjusting LDSC heritability estimates through sample size modifications **19**

8. The effects of selection on the associations between trait and genotypes **19**

References **21**

Supplementary Tables

	TNTC		WSPC		
**Quantitative**	**Effect**	***P*-value**	**Effect**	***P*-value**	**N**
Educational attainment	0.0104	2.5E-07	0.0244	1.4E-33	260,950
Age at first birth (women)	0.0093	3.7E-03	0.0208	1.0E-10	98,653
BMI	-0.0114	5.3E-09	-0.0081	3.6E-05	271,535
HDL cholesterol	0.0034	0.11	0.0058	6.7E-03	237,785
Height	0.0033	0.11	0.0083	5.1E-05	271,820
Glycated haemoglobin	-0.0074	2.6E-04	0.0004	0.83	259,594
Number of siblings	-0.0043	3.3E-02	-0.0046	2.4E-02	268,191
Number of children	-0.0011	0.56	-0.0034	8.3E-02	271,317
Glucose	-0.0010	0.64	-0.0047	2.3E-02	237,629
Vitamin D	-0.0007	0.75	-0.0018	0.37	249,079
Sex hormone binding globulin	0.0072	6.0E-04	0.0019	0.37	235,960
Grip strength	-0.0023	0.24	0.0011	0.57	270,525
Lipoprotein A	-0.0021	0.29	-0.0034	0.10	251,698
Testosterone	0.0031	0.12	0.0022	0.26	257,570
**Binary**	**log(OR)**	***P*-value**	**log(OR)**	***P*-value**	**Ncase/Ncontrol**
Dietary study invitation	0.0103	1.4E-02	0.0261	6.4E-10	166,993/105,416
Dietary study participation	0.0153	3.6E-03	0.0126	1.7E-02	54,124/112,869
Physical activity study invitation	0.0153	8.8E-05	0.0187	1.7E-06	132,633/139,776
Physical activity study participation	0.0183	1.0E-03	0.0221	6.8E-05	59,455/73,178

Supplementary Table 1: Unadjusted TNTC and WSPC PGS associations with phenotypes. Association results here correspond to those in Table 1 for *pPGS*s based on TNTC and WSPC. The difference is that the weights used here are not adjusted for the major allele bias. *P*-values (*t*-tests for quantitative phenotypes and *z*-tests for binary phenotypes) are two-sided without multiple comparison correction.

**Phenotype**	***ρ***	**(se)**
Educational attainment	0.419	(0.132)
Age at first birth	0.422	(0.148)
BMI	-0.127	(0.063)
Dietary study invitation	0.493	(0.169)
Dietary study participation	0.184	(0.128)
Physical activity study invitation	0.423	(0.165)
Physical activity study participation	0.479	(0.190)

Supplementary Table 2: LD score regression estimates of genetic correlations between primary participation and traits using WSPC results.
Results correspond to those in Table 2. The difference is that the genetic correlations (ρ) between primary participation and the traits are estimated here using results from WSPC instead of BSPC (see Methods).

Phenotype	h^2	(se)
Primary Participation (BSPC)	0.125	(0.044)
Dietary study invitation	0.060	(0.004)
Dietary study participation	0.062	(0.005)
Physical activity study invitation	0.034	(0.003)
Physical activity study participation	0.046	(0.006)

Supplementary Table 3. Heritability estimates for liability scores underlying participation traits. Heritability estimates for the liability scores underlying participation traits. LD score regression using the program LDSC was used plus adjustments described in Methods. See also Supplementary Note Section 7.

Supplementary Note

1. Identifying relatives

The UKBB data includes kinship coefficients, attained with the program KING[1], for pairs that were genetically inferred to be related of 3rd degree or closer. Among those pairs, we identified first-degree relatives with WB descent, 16,668 sibling pairs and 4,427 parent-offspring pairs, using kinship coefficient boundaries recommended by the authors of KING[1]. Pairs with kinship coefficient >0.177 and <0.354 were concluded to be first degree relatives and, following Bycroft et al. (2018)[2], those who shared less than 0.12% IBD0, were classified as parent-offspring pairs

These two groups, siblings and parent-offspring pairs, did not overlap. That is, none of the sibling pairs had a parent present in the dataset and none of the parent-offspring pairs had a sibling present in the dataset. The parent-offspring pairs were made up of 739 trios, 635 father-offspring pairs and 2,314 mother-offspring pairs. The age-difference between the parents and their offspring ranged from 15 to 31 years with the mean being 23.43 years (SD=2.71). For sibling-groups with more than 2 siblings in the dataset, we chose the first two participating siblings in the group.

Within the WB subset, 272,409 individuals had no close relatives within the UKBB (>3rd degree for all pairs). We refer to this set of individuals as the 'unrelated' set. The male fraction was 46.7% in the 'unrelated' set compared to 31.0% among the parents and 42.4% among the siblings.

2. *t*-statistics, standard errors, and sample sizes

For a particular sequence variant, let n_{PO}, n_0, n_1, n_2, be respectively the number of parent-offspring pairs, the number of IBD0 sib-pairs, the number of IBD1 sib-pairs, and the number of IBD2 sib-pairs. For TNTC, let T_i and NT_i be respectively the transmitted and non-transmitted allele for parent-offspring pair $i, i = 1, \ldots, n_{po}$. Let

$$DTNT_i = T_i - NT_i \tag{20}$$

It is easy to see that the sample mean $\overline{DTNT}$ is equivalent to $F_T - F_{NT}$, expression (1). Its SE is calculated as

$$\frac{s_{DTNT}}{\sqrt{n_{PO}}} \tag{21}$$

where s_{DTNT} is the sample standard deviation of the $DTNT_i$s. Similarly, for WSPC, let $S_{i,}$ NS_{i1} and NS_{i2} be respectively the shared and two and not-shared alleles for IBD1 sib-pair $i, i = 1, \dots, n_1$. Let

$$DSNS_i = S_i - \frac{(NS_{i1} + NS_{i2})}{2} \tag{22}$$

The sample mean $\overline{DSNS}$ is equivalent to $F_S - F_{NS}$, expression (2). Its SE is calculated as

$$\frac{s_{DSNS}}{\sqrt{n_1}} \tag{23}$$

where s_{DSNS} is the sample standard deviation of the $DSNS_i$s. For BSPC, let F_{0i} be the frequency of allele 1 in IBD0 sib-pair $i, i = 1, \dots, n_o$, and let F_{2j} be the frequency of allele 1 in IBD2 sib-pair $j, j = 1, \dots, n_2$. Let $\overline{F_0}$ and $\overline{F_2}$ be the corresponding sample means. Expression (3), or $F_{IBD2} - F_{IBD0}$, is equivalent to $\overline{F_2} - \overline{F_0}$. Its SE can be calculated as

$$\sqrt{\frac{s_2^2}{n_2} + \frac{s_0^2}{n_0}} \tag{24}$$

where s_2^2 is the sample variance of the F_{2j}s, and s_0^2 is the sample variance of the F_{0i}s. Note that F_{0i} is the average of 4 distinct alleles in the IBD sense, while F_{2j} is the average of two distinct alleles. Hence s_2^2 is approximately two times s_0^2.

In the current study, the TNTC statistics were based on 4,427 parent-offspring pairs for all sequence variants. The sample size for the sibling specific *t*-statistics varied between sequence variants due to the variation in IBD sharing and because of the trimming procedure described above. In the subset of the 500,632 high-quality sequence variants, the sample size for computing the WSPC statistics ranged from 6,428 sibling pairs to 8,315 sibling pairs (mean=7,512; SD=279). The sample size for computing the IBD=2 specific allele frequencies

ranged from 3,137 to 4,246 sibling pairs (mean=3,763; SD=159) while the sample size for computing the IBD=0 specific allele frequencies ranged from 3,110 to 4,202 sibling pairs (mean=3,723; SD=143).

3. The major allele bias

A summary about the causes of the major allele bias and solutions:

(i) The errors are not due to violations of the principles we put forward about how participation bias would leave footprints in the sample. They are also not related to sampling issues such as population stratification. Instead, they are consequences of data (genotyping) and data processing (IBD estimation and phasing) errors.

(ii) The IBD estimation problem is essentially resolved by using the recently available software *snipar* and trimming of SNP genotypes at the edges of the estimated IBD regions. Consequentially, the BSPC results are now very clean. In the set of 500,632 high-quality sequence variants, the correlation between the BSPC *t*-statistics and the frequency of allele 1 is 0.0075 ($r^2 = 5.6 \times 10^{-5}$). Even though still statistically significant from zero, this correlation is very small. The small correlation observed for BSPC could be mostly 'real' as opposed to being a bias induced by errors. Firstly, there is no reason to believe that the effects of the major and minor alleles would be perfectly balanced within this set of sequence variants. Secondly, and most interestingly, if we assume the effects of the major and minor alleles are perfectly balanced in the population, alleles that have a higher frequency in the sample would actually have a slightly higher chance of having a positive participation effect than alleles that have a lower frequency. This is because selection would increase the frequency of an allele with positive participation effect relative to the population. An obvious example would be a biallelic sequence variant for which both alleles are of 50% frequency in the population. The participation promoting allele would have a frequency higher than 50% in the sample, and appears to be the major allele. Thirdly, when LD score regression is applied to the χ^2 statistics computed from the BSPC *t*-statistics, the fitted intercept is 0.9998 (SE=0.0053), practically equal to one. Most importantly, this supports not only that the current BSPC *t*-statistics are minimally affected by data and data processing artefacts, they are also not affected by the usual confounding factors such as population stratification, a distinct property of the proposed method.

(iii) Genotyping errors and phasing errors affect TNTC and WSPC through the step that a genotype is split into a shared and a not-shared allele. Investigations, including simulations, show that 'random' genotyping and phasing errors would tend to lead to a major allele bias. By estimating the phasing error rate using genotyped trios, we estimate that it can account for approximately one-half of the observed major allele bias for SNPs with MAF > 0.1. For SNPs with MAF < 0.1, particular those with MAF close to 0.01, we believe that genotyping errors are responsible for a higher fraction of the major allele bias. Additional quantitative details are given at the end of this section.

(iv) Two-step adjustment applied to the TNTC and WSPC test statistics. The first adjustment is to shift the *t*-statistics towards the minor allele to eliminate the major allele bias when the SNPs are examined as a group. This step, however, cannot remove all the bias for the SNPs on an individual level. That is because this adjustment is based on allele frequency, but the bias varies within a group of SNPs with similar allele frequencies. That variation may be modest for the phasing induced bias, but likely to be more substantial for the genotyping-error induced bias. This bias variation within SNPs with similar MAF leads to an inflation of the average χ^2 values. This inflation is observed to be substantially higher for SNPs with low MAF, consistent with the belief that genotyping errors play a more important role there. Because the inflation is negatively correlated with MAF and MAF is positively correlated with LD scores, applying LD score regression to these χ^2 values would lead to highly misleading results. To address this problem, we applied a MAF-specific genomic control adjustment to the χ^2 values, which is equivalent to shrinking the corresponding *z*-scores towards 0.

(v) The effect of the major allele bias depends on the analysis. Firstly, and most importantly, the BSPC results are currently not affected by the major allele bias. For TNTC and WSPC, the major allele bias has only minor impact on polygenic score prediction as there it just amounts to adding a small amount of noise. This is supported by the similarities of the association results obtained using polygenic scores constructed with and without adjustments. For LD score regressions, the adjustments are important for getting reasonable, if not perfect results. For evaluating statistical significance of individual SNPs, we belief that the TNTC and WSPC association results with adjustments can be used to support/validate associations observed with BSPC. In addition, with the former, there are less concerns with common SNPs. For

SNPs that have low MAFs and positive associations with the major allele are observed for TNTC and WSPC, consistent result observed with BSPC is needed for the association to be taken seriously.

Further comments on results displayed in Extended Data Figure 5. The results show that the error rate is around 0.5% when $f < 0.5$, but becomes substantially higher when f is close to 1. For example, ε_f is estimated as 1.44% and 0.50% for f equal to 0.9 and 0.1 respectively. Despite that, the bias $f\varepsilon_{1-f} - (1-f)\varepsilon_f$ remains positive for $f = 0.9$ as $(1.44/0.50) = 2.88 < (.9/.1) = 9$. In general, the bias is positive for $f >$ 0.5 and negative for $f < 0.5$.

Further comments on results displayed in Extended Data Figure 6. In Extended Data Figure 6, displayed (solid line) is the estimated bias of the WSPC *t*-statistics as a function of f, frequency of allele 1 in the sample. Let cf be $(f - 0.5)$. The estimated bias is calculated by regressing the *t*-statistics of the 500,632 SNPs, through the origin, on cf and cf^3. Regressing through the origin and the odd powers of cf is to ensure that these results are invariant to which allele of a SNP is designated as allele 1, i.e. the estimated bias for $f = a$ is negative of the estimated bias for $f = (1 - a)$. We note that including the cf^3 term provide a slightly better fit, although the improvement is very small compared to only regressing on cf. In the same figure, also displayed (broken line) is estimated bias induced by miscalling the shared allele for the double-heterozygotes, calculated as follows. Because the chance of double-heterozygotes is $f(1-f)$ under the simplifying assumption of random mating and there is a corresponding bias to the not-shared allele with an opposite sign, the bias of $(F_{IBD1S} - F_{IBD1NS})$ is $2f(1-f)[f\varepsilon_{1-f} - (1-f)\varepsilon_f]$. The induced bias to the *t*-statistic is the latter divided by the estimated standard error of $(F_{IBD1S} - F_{IBD1NS})$. Note again that this bias is 0 for $f = 0.5$, negative for $f < 0.5$, and positive for $f > 0.5$. Moreover, it is invariant to which of the two alleles of a SNP is designated as allele 1 as the bias for $f = a$ is negative of the bias for $f = (1 - a)$. There is clearly a non-linear component, which simply is the mathematical behaviour of this bias.

The Nature of the genotyping-error induced major allele bias. While the genotyping-error mechanism is in general complicated and varies between SNPs, the main driving force of the major allele bias for SNPs with low *MAF*s can be understood as follows. When allele 1 is the major allele with a high frequency, the overall bias is dominated by the bias induced when a true genotype 2 is being miscalled 1 for one sibling. In this case, most of the time the genotype of the other sibling would be 2, *i.e.* the shared allele and the two not-shared alleles

are all 1 in reality. With the miscalled genotype, while the shared allele would still be correctly determined as allele 1, the two 'observed' not-shared alleles would consist of one 1 allele and one 0 allele. Thus, in this scenario, the induced positive bias on $(F_{IBD1S} - F_{IBD1NS})$ is through overcalling the minor allele 0 in the not-shared alleles, as opposed to overcalling the major allele 1 in the shared alleles. It is noted that for SNPs with high allele 1 frequencies, for similar reasons, when a true genotype 0 is being miscalled 1 for one sibling, it tends to induce a negative bias on $(F_{IBD1S} - F_{IBD1NS})$. However, since 0 genotypes have much lower frequencies than genotypes 2 for these SNPs, the negative bias is not enough to cancel out the positive bias unless the 0 genotypes are much more frequently miscalled relative to the 2 genotypes.

4. Additional quality filtering of phased SNPs

To reduce the impact of data artefacts on association analysis, we restricted the primary participation GWAS results to sequence variants in the UKBB haplotype data that fulfilled the following additional quality control criteria:

- Did not deviate significantly from HWE at $P < 10^{-5}$ in a group of 272,234 WB individuals with no close relatives in UKBB (exact test with PLINK 1.90).
- Not among the 250 sequence variants in the beginning and end of each chromosome.
- Not in extended LD regions as reported by Price et al. (2008).[3]
- With *MAF*>1% in the set of 16,668 sibling pairs
- With a rsname
- With non-ambiguous nucleotides
- Did not deviate significantly from shared allele expectations at *P*< 10^{-6} in the group of double heterozygous parent-offspring pairs. Specifically, for each SNP, we investigated the accuracy of the phasing provided by UKBB[2] by comparing the expected fraction of the shared allele to the observed fraction of the shared allele in the group of parent-offspring pairs that were heterozygous for the variant in question. A heterozygous parent passes either allele, 0 or 1, with an equal probability to his/her offspring. If the offspring is heterozygous as well, the probability of the shared allele being the one coded as 1 is simply the probability of the allele inherited from the other parent being the one coded as 0. Assuming random mating, the allele inherited

from the other parent can be regarded as a randomly drawn allele from the population and hence, in the double heterozygous scenario, the probability of the shared allele being the one coded as 1 is the population allele frequency of the allele coded as 0. We removed 262 sequence variants for which the observed fraction of the shared allele, among the double heterozygous parent-offspring pairs, deviated from the expected fraction at a threshold of $P<10^{-6}$ (two-sided binomial test). Of those, 79 sequence variants deviated from HWE with $P<10^{-5}$ (exact test).

- Observed fraction of sibling pairs sharing the sequence variant IBD 0, 1 or 2, no more than 3 standard deviations above the expected fraction, 0.25, 0.5 and 0.25 respectively. Note that if one IBD status (0, 1 or 2) is overcalled then at least one of the two other IBD statuses is undercalled for that specific variant. Hence, thresholding the IBD fractions from above also affects the lower bounds.
- Had missing rate below 5% among the 16,668 sibling pairs in the genotyping array data.

After applying these additional filters, 500,632 sequence variants remained in the primary participation genome scan. The genetic variants in the UKBB haplotype data had previously been filtered by Bycroft et al. (2018) based on various quality control criteria[2], including deviations from HWE. As the current analysis involves applying a new method to the UKBB data, an additional HWE filter was applied here.

It is noted that the missing rate filter is not based on the phased haplotype data, in which there are no missing genotypes, but the genotype array data from which the haplotypes were estimated from. The reason behind this filter is that the array genotypes are not missing at random and a higher rate of missingness can be associated with lower data quality overall. All the genetic variants in the UKBB haplotype data have a missing rate below 5% in the genotyping array data in a set of 463,844 individuals within the UKBB dataset[2]. However, as non-random missingness and potentially lower quality genotypes could have significant impact for certain analysis, we further omitted sequence variants that have a missing rate above 5% in the subsample of 16,668 sibling pairs.

5. Phenotypes

By using various data-fields in the UKBB data release, we constructed a range of phenotypic variables for the 272,409 WB ‘unrelated’ individuals. The phenotypes of interest were

adjusted for year of birth (data-field 34), age at recruitment (data-field 21022), genotyping array (data-field 22000), and sex (data-field 31) when applicable. We accounted for population stratification by using principal components (PCs) which were inferred with the program ProPCA[4]. Details about those PCs are described in a previous publication[5]. Quantitative phenotypes were rank-based inverse normalised[6] except for educational attainment as it is not approximately normally distributed. Details about phenotype specific protocols and adjustments are provided below.

5.1 Educational attainment

We constructed an educational attainment variable from the data-fields 'Qualifications' (data-field 6138) and 'Age completed full-time education' (data-field 845). Both of these data-fields contain answers to questions that participants were asked on a touch-screen in the initial assessment visit. The data-field 'Qualifications' denotes answers to the multiple-choice question: 'Which of the following qualifications do you have? (You can select more than one).' We mapped the multiple-choice options to years of schooling using the ISCED classification[7] (see below), except for the option 'NVQ or HND or HNC or equivalent'. Following Okbay et al. (2022), years of schooling of NVQ/HND/HNC holders was defined to be their answer in the data-field 'Age completed full-time education' minus five[8]. Individuals that answered 'Prefer not to answer' were mapped to 'Not applicable' and were excluded from subsequent analysis.

1) College or University degree: 20 years
2) A levels/AS levels or equivalent: 13 years
3) O levels/GCSEs or equivalent: 10 years
4) CSEs or equivalent: 10 years
5) NVQ or HND or HNC or equivalent: (Age completed full time education – 5) years
6) Other professional qualifications eg: nursing, teaching: 15 years
7) None of the above: 7 years
8) Prefer not to answer: Not applicable

Respondents who selected multiple options were assigned their highest category (maximum years of schooling). Individuals who had maximum year of schooling below 7 years were excluded from subsequent analysis.

In the subset of 260,950 WB ‘unrelated’ individuals with applicable entries for year-of-schooling, we adjusted for year of birth up to the order of three, age at recruitment and 40 PCs separately for each sex. This was done with a linear regression in R where years-of-schooling equivalent was treated as a quantitative dependent variable. The mean centred and standardised residuals from these two regressions (men and women) were
used as an approximation for educational attainment in subsequent analysis.

5.2 Age at first birth (AFB)

The AFB variable was constructed from answers to the touchscreen question ‘How old were you when you had your FIRST child?’ which was asked in the initial assessment visit, (data-field 2754: ‘Age at first live birth’). This information was collected from women who had indicated that they had given birth to a child in an answer to a previous question in the touchscreen questionnaire (data-field 2734: ‘Number of live births’). We excluded entries below 12. In the subset of 98,653 WB ‘unrelated’ women with applicable AFB entries, we adjusted for year of birth up to the order of three, age at recruitment and 40 PCs by performing linear regression in R. The resulting residuals were then rank-based inverse normalised.

5. 3 Body mass index (BMI)

The BMI variable was based on the data-field 21001-0-0: ‘Body Mass index (BMI)’, which was computed from height and weight measurements recorded in the initial assessment visit. In the subset of 271,535 WB ‘unrelated’ individuals with BMI values, we adjusted for year of birth, age at recruitment up to the order of three and 40 PCs separately for each sex by performing linear regression in R treating rank-based inverse normalised BMI values as a dependent variable. The resulting residuals, attained for males and females separately, were then rank-based inverse normalised.

5.4 High-Density-Lipoprotein (HDL) cholesterol

The HDL cholesterol variable was based on data-field 30760-0-0: ‘HDL Cholesterol’, a biochemistry marker measured in blood samples collected at recruitment. In the group of WB ‘unrelated’ individuals, 34,628 individuals had missing HDL cholesterol values and, as indicated by data-field 30765: ‘HDL cholesterol missing reason’ and data-field 30766: ‘HDL cholesterol missing reportability’, 3 of those were missing because the values were below the reportable range and one was above the reportable range. We imputed the ‘below reportable range’ entries to the minimum reportable HDL cholesterol measurement in the group of WB ‘unrelated’ individuals minus 0.001, that is to 0.218, and the ‘above reportable range’ entry to

the maximum reportable HDL cholesterol measurement plus 0.001, that is to 4.402. Then, in the group of 237,785 WB ‘unrelated’ individuals with applicable values, we adjusted the HDL cholesterol values for year of birth, age at recruitment up to the order of three and 40 PCs separately for each sex by performing linear regression in R treating rank-based inverse normalised HDL cholesterol values (including the imputed ones) as a dependent variable. The resulting residuals, attained for males and females separately, were then rank-based inverse normalised.

5.5 Height

The height variable was based on data-field 50-0-0: ‘Standing height’, which was measured in the initial assessment visit. In the subset of 271,820 WB ‘unrelated’ individuals with height measurements, we adjusted for year of birth, age at recruitment up to the order of three and 40 PCs separately for each sex by performing linear regression in R treating rank-based inverse normalised height values as a dependent variable. The resulting residuals, attained for males and females separately, were then rank-based inverse normalised.

5.6 Glycated haemoglobin (HbA1c)

The HbA1c variable was based on data-field 30750-0-0: ‘Glycated haemoglobin (HbA1c)’, a biochemistry marker measured in blood samples collected at recruitment. In the group of WB ‘unrelated’ individuals, 12,926 individuals had missing HbA1c values. As indicated by data-field 30755: ‘Glycated haemoglobin (HbA1c) missing reason’ and data-field 30756 ‘Glycated haemoglobin (HbA1c) reportability’, 111 of those were missing because the values were below reportable range. We imputed the ‘below reportable range’ entries to the minimum HbA1c measurement minus 0.001, that is to 15.299. In the group of 259,594 WB ‘unrelated’ individuals with applicable values, we adjusted HbA1c levels for year of birth, age at recruitment up to the order of three and 40 PCs separately for each sex by performing linear regression in R treating rank-based inverse normalised HbA1c values (including the imputed ones) as a dependent variable. The resulting residuals, attained for males and females separately, were then rank-based inverse normalised.

5.7 Number of siblings (N. siblings)

The N. siblings variable was constructed from answers to the touchscreen questions ‘How many sisters do you have? (Please include those who have died, and twin sisters. Do not include half-sisters, step-sisters or adopted sisters)’ (data-field 1883: ‘Number of full sisters’) and ‘How many brothers do you have? (Please include those who have died, and twin brothers. Do not include half-brothers, step-brothers or adopted brothers)’ (data-field 1873:

‘Number of full brothers’). Both questions were asked in the initial assessment visit. In the subset of 268,191 WB ‘unrelated’ individuals with applicable N. siblings entries, we adjusted for year of birth, age at recruitment up to the order of three and 40 PCs separately for each sex by performing linear regression in R. The resulting residuals, attained for males and females separately, were then rank-based inverse normalised.

5.8 Number of children (N. children)

The N. children variable was constructed from answers to the touchscreen questions ‘How many children have you fathered?’ (data-field 2405: ‘Number of children fathered’) and ‘How many children have you given birth to? (Please include live births only)’ (data-field 2734: ‘Number of live births’). Both questions were asked in the initial assessment visit. Data-field 2405 was only collected from males and data-field 2734 was only collected from women. In the subset of 271,317 WB ‘unrelated’ individuals with applicable N. children entries, we adjusted for year of birth up to the order of three, age at recruitment and 40 PCs separately for each sex by performing linear regression in R. The resulting residuals, attained for males and females separately, were then rank-based inverse normalised.

5.9 Glucose

The glucose variable was based on data-field 30740-0-0: ‘Glucose’, a biochemistry marker measured in blood samples collected at recruitment. In the group of WB ‘unrelated’ individuals, 34,783 individuals had missing glucose values. As indicated by data-field 30745: ‘Glucose missing reason’ and data-field 30746 ‘Glucose reportability’, three of those were missing because the values were below reportable range. We imputed the ‘below reportable range’ entries to the minimum glucose measurement minus 0.001, that is to 1.004. In the group of 237,629 WB ‘unrelated’ individuals with applicable values, we adjusted glucose levels for year of birth, age at recruitment up to the order of three and 40 PCs separately for each sex by performing linear regression in R treating rank-based inverse normalised glucose values (including the imputed ones) as a dependent variable. The resulting residuals, attained for males and females separately, were then rank-based inverse normalised.

5.10 Vitamin D

The vitamin D variable was based on data-field 30890-0-0: ‘Vitamin D’, a biochemistry marker measured in blood samples collected at recruitment. In the group of WB ‘unrelated’ individuals, 24,116 individuals had missing vitamin D values. As indicated by data-field 30895: ‘Vitamin D missing reason’ and data-field 30896 ‘Vitamin D reportability’, 784 of those were missing because the values were below reportable range and 2 were missing

because the values were above the reportable range. We imputed the ‘below reportable range’ entries to the minimum vitamin D measurement minus 0.001, that is to 9.999 and the ‘above reportable range’ entries to the maximum vitamin D measurement plus 0.001, that is to 340.001. In the group of 249,079 WB ‘unrelated’ individuals with applicable values, we adjusted vitamin D levels for year of birth, age at recruitment up to the order of three and 40 PCs separately for each sex by performing linear regression in R treating rank-based inverse normalised vitamin D values (including the imputed ones) as a dependent variable. The resulting residuals, attained for males and females separately, were then rank-based inverse normalised.

5.11 Sex hormone binding globulin (SHBG)

The SHBG variable was based on data-field 30830-0-0: ‘SHBG’, a biochemistry marker measured in blood samples collected at recruitment. In the group of WB ‘unrelated’ individuals, 36,843 individuals had missing SHBG values. As indicated by data-field 30835: ‘SHBG missing reason’ and data-field 30836 ‘SHBG reportability’, 5 of those were missing because the values were below reportable range and 389 were missing because the values were above the reportable range. We imputed the ‘below reportable range’ entries to the minimum SHBG measurement minus 0.001, that is to 0.389 and the ‘above reportable range’ entries to the maximum SHBG measurement plus 0.001, that is to 241.921. In the group of 235,960 WB ‘unrelated’ individuals with applicable values, we adjusted SHBG levels for year of birth, age at recruitment up to the order of three and 40 PCs separately for each sex by performing linear regression in R treating rank-based inverse normalised SHBG values (including the imputed ones) as a dependent variable. The resulting residuals, attained for males and females separately, were then rank-based inverse normalised.

5.12 Grip strength

The grip strength variable was based on data-field 46 ‘Hand grip strength (left)’ and data-field 47 ‘Hand grip strength (right)’, physical measures attained in the initial assessment visit. For each individual we chose the maximum value (left, right) as their grip strength measurement. For the 270,525 WB ‘unrelated’ individuals with non-missing values, we adjusted grip strength measurements for year of birth, age at recruitment up to the order of three, standing height and 40 PCs separately for each sex by performing linear regression in R treating rank-based inverse normalised grip strength values as a dependent variable. The resulting residuals, attained for males and females separately, were then rank-based inverse normalised.

5.13 Lipoprotein A

The lipoprotein A variable was based on data-field 30790-0-0: ‘Lipoprotein A’, a biochemistry marker measured in blood samples collected at recruitment. In the group of WB ‘unrelated’ individuals, 65,941 individuals had missing lipoprotein A values. As indicated by data-field 30795: ‘Lipoprotein A missing reason’ and data-field 30796 ‘Lipoprotein A reportability’, 26,943 of those were missing because the values were below reportable range and 18,287 were missing because the values were above the reportable range. We imputed the ‘below reportable range’ entries to the minimum lipoprotein A measurement minus 0.001, that is to 3.799 and the ‘above reportable range’ entries to the maximum lipoprotein A measurement plus 0.001, that is to 189.001. In the group of 251,698 WB ‘unrelated’ individuals with applicable values, we adjusted lipoprotein A levels for year of birth, age at recruitment up to the order of three and 40 PCs separately for each sex by performing linear regression in R treating rank-based inverse normalised lipoprotein A values (including the imputed ones) as a dependent variable. The resulting residuals, attained for males and females separately, were then rank-based inverse normalised.

5.14 Testosterone

The testosterone variable was based on data-field 30850-0-0: ‘Testosterone’, a biochemistry marker measured in blood samples collected at recruitment. In the group of WB ‘unrelated’ individuals, 36,861 individuals had missing testosterone values. As indicated by data-field 30855: ‘Testosterone missing reason’ and data-field 30856: ‘Testosterone reportability’, 22,009 of those were missing because the values were below reportable range and 13 were missing because the values were above the reportable range. We imputed the ‘below reportable range’ entries to the minimum testosterone measurement minus 0.001, that is to 0.349 and the ‘above reportable range’ entries to the maximum testosterone measurement plus 0.001, that is to 54.343. In the group of 257,570 WB ‘unrelated’ individuals with applicable values, we adjusted testosterone levels for year of birth, age at recruitment up to the order of three and 40 PCs separately for each sex by performing linear regression in R treating rank-based inverse normalised testosterone values (including the imputed ones) as a dependent variable. The resulting residuals, attained for males and females separately, were then rank-based inverse normalised.

5.15 Dietary study invitation

The dietary study invitation variable was based on data-field 110001: ‘Invitation to complete online 24-hour recall dietary questionnaire, acceptance’. The 166,993 WB ‘unrelated’

individuals with the data-field values ‘No response’, ‘Partial’ or ‘Completed’ were defined as cases. That is, they had been invited to participate in the study. The 105,416 WB ‘unrelated’ individuals with non-applicable (NA) values in this field were defined as controls. That is, they had not been invited to the study.

5.16 Dietary study participation

The dietary study participation variable was based on data-field 110001: ‘Invitation to complete online 24-hour recall dietary questionnaire, acceptance’. The 54,124 WB ‘unrelated’ individuals with the values ‘Partial’ or ‘Completed’ in the field were defined as cases. The 112,869 WB ‘unrelated’ individuals with the data-field value ‘No response’ were defined as controls.

5.17 Physical activity study invitation

The physical activity study invitation variable was based on data-field 110005: ‘Invitation to physical activity study, acceptance’. The 132,633 WB ‘unrelated’ individuals with the data-field values ‘No response’, ‘Partial’ or ‘Completed’ were defined as cases. That is, they had been invited to participate in the study. The 139,776 WB ‘unrelated’ individuals with non-applicable (NA) values in this field were defined as controls. That is, they had not been invited to the study.

5.18 Physical activity study participation

The physical activity study participation variable was based on data-field 110005: ‘Invitation to physical activity study, acceptance’. The 59,455 WB ‘unrelated’ individuals with the data-field values ‘Partial’ or ‘Completed’ were defined as cases. The 73,178 WB ‘unrelated’ individuals with the data-field value ‘No response’ were defined as controls.

6. Genetic components captured by TNTC, WSPC and BSPC

As noted in the main text, the participation bias effects captured by the three comparisons would in general be a combination of ‘voluntary’ and ‘involuntary’ effects. The involuntary component, in particular, can depend highly on the sampling scheme. WSPC and BSPC are based on the same set of participating sibling pairs, *i.e.* in general a sibling pair would be IBD0, IBD1, and IBD2 at different sites in the genome. As demonstrated by the simulations (Figure 4), under an additive model and assuming variants have small effects individually, WSPC and BSPC would be capturing very similar effects, *i.e.* the correlation of the effects they captured across all the variants would be close to 100%. Deviations from the additive model for some variants can reduce that correlation somewhat, but it is not expected to be

substantial. By contrast, the effects captured by TNTC can be more different from that captured by WSPC/BSPC as it is based on a different set of participating individuals. In particular, the parents are substantially older than the offspring and thus the reasons underlying the participation of the parents can be quite different from that driving the participation of the offspring. In general, how different TNTC and WSPC/BSPC are depends on the specific study, while WSPC and BSCP are expected to be very similar, with respect to true effects captured, for all studies. For UKBB, the average year of birth of the sibling pairs (1950.8) is very close to that of the WB sample as a whole (1951.2). By contrast, the parents of the parent-offspring pairs have a much earlier average (1941.7), and the offspring have a much later average (1965.0). Adding this to having larger sample size and being insensitive to data artefacts, genetic correlation and heritability estimates based on BSPC are much more relevant than estimates based on TNTC.

7. Adjusting LDSC heritability estimates through sample size modifications

As highlighted by equation (11) in Methods, the association between a genetic component, or the genotype of an individual SNP, is in general weaker, or statistically less efficient, with I than with X. This means that, for a fixed set of χ^2 statistics, the estimated heritability is inversely proportional to the assumed efficiency of the tests. Indeed, instead of obtaining the estimated heritability of X by multiplying an adjustment factor to the estimate from LDSC, we can also simply provide LDSC with an adjusted/reduced sample size. For example, for the Dietary Study invitation, by entering a sample size of $272{,}409 \times (0.786)^2$, rounded to 168,293, the LDSC program will give the estimate of 0.060 directly, the same as (13) in Methods. Here, 168,293 can be thought of as the effective sample size. The principle that the adjustment factor is inversely proportional to the statistical efficiencies of the tests is also relevant to deriving an estimate for the heritability of primary participation.

8. The effects of selection on the associations between trait and genotypes

Suppose there is biased sample selection with respect to a trait Y, directly or indirectly through a correlated trait. The type of biased selection studied here usually would decrease the sample variance of Y. If Y is correlated with a genotype g in the population, it is rather common knowledge that r^2 between Y and g would be reduced in the sample. What is not quite common knowledge is that biased sample selection can create artificial epistatic effects for variants that have an effect on the trait. For example, suppose the relationship between trait Y and the genotypes of two variants, g_1 and g_2, is additive in the population:

$$Y = a_1 g_1 + a_2 g_2 + noise.$$

Suppose, a_1 and a_2 are both positive. In the biased sample, not only would the expected values of the fitted coefficients for g_1 and g_2 shrink, the expected value of the fitted coefficient for the interaction term $g_1 g_2$ would be non-zero and positive. This can be easily confirmed by simulations.

References

1 Manichaikul, A. *et al.* Robust relationship inference in genome-wide association studies. *Bioinformatics* **26**, 2867-2873, doi:10.1093/bioinformatics/btq559 (2010).

2 Bycroft, C. *et al.* The UK Biobank resource with deep phenotyping and genomic data. *Nature* **562**, 203-209, doi:10.1038/s41586-018-0579-z (2018).

3 Price, A. L. *et al.* Long-range LD can confound genome scans in admixed populations. *Am J Hum Genet* **83**, 132-135; author reply 135-139, doi:10.1016/j.ajhg.2008.06.005 (2008).

4 Agrawal, A., Chiu, A. M., Le, M., Halperin, E. & Sankararaman, S. Scalable probabilistic PCA for large-scale genetic variation data. *PLoS genetics* **16**, e1008773, doi:10.1371/journal.pgen.1008773 (2020).

5 Young, A. I., Benonisdottir, S., Przeworski, M. & Kong, A. Deconstructing the sources of genotype-phenotype associations in humans. *Science* **365**, 1396-1400, doi:10.1126/science.aax3710 (2019).

6 Beasley, T. M., Erickson, S. & Allison, D. B. Rank-based inverse normal transformations are increasingly used, but are they merited? *Behavior genetics* **39**, 580-595, doi:10.1007/s10519-009-9281-0 (2009).

7 Lee, J. J. *et al.* Gene discovery and polygenic prediction from a genome-wide association study of educational attainment in 1.1 million individuals. *Nature Genetics* **50**, 1112-1121, doi:10.1038/s41588-018-0147-3 (2018).

8 Okbay, A. *et al.* Polygenic prediction of educational attainment within and between families from genome-wide association analyses in 3 million individuals. *Nature genetics* **54**, 437-449, doi:10.1038/s41588-022-01016-z (2022).
