# Supplementary material for: Studying the genetics of participation using footprints left on the ascertained genotypes
Source: Nat Genet. 2023 Jul 13;55(8):1413–20. doi: 10.1038/s41588-023-01439-2 (PMC10412458; doi:10.1038/s41588-023-01439-2)
Supplement: Supplementary file 2 — Reporting Summary [file 41588_2023_1439_MOESM2_ESM.pdf]

nature portfolio

Corresponding author(s): Stefania Benonisdottir
Augustine Kong

Last updated by author(s): May 26, 2023

Reporting Summary

Nature Portfolio wishes to improve the reproducibility of the work that we publish. This form provides structure for consistency and transparency in reporting. For further information on Nature Portfolio policies, see our Editorial Policies and the Editorial Policy Checklist.

Statistics

For all statistical analyses, confirm that the following items are present in the figure legend, table legend, main text, or Methods section.

n/a	Confirmed	
☐	☒	The exact sample size (*n*) for each experimental group/condition, given as a discrete number and unit of measurement
☒	☐	A statement on whether measurements were taken from distinct samples or whether the same sample was measured repeatedly
☐	☒	The statistical test(s) used AND whether they are one- or two-sided<br>*Only common tests should be described solely by name; describe more complex techniques in the Methods section.*
☐	☒	A description of all covariates tested
☐	☒	A description of any assumptions or corrections, such as tests of normality and adjustment for multiple comparisons
☐	☒	A full description of the statistical parameters including central tendency (e.g. means) or other basic estimates (e.g. regression coefficient) AND variation (e.g. standard deviation) or associated estimates of uncertainty (e.g. confidence intervals)
☐	☒	For null hypothesis testing, the test statistic (e.g. *F*, *t*, *r*) with confidence intervals, effect sizes, degrees of freedom and *P* value noted<br>*Give P values as exact values whenever suitable.*
☒	☐	For Bayesian analysis, information on the choice of priors and Markov chain Monte Carlo settings
☒	☐	For hierarchical and complex designs, identification of the appropriate level for tests and full reporting of outcomes
☒	☐	Estimates of effect sizes (e.g. Cohen's *d*, Pearson's *r*), indicating how they were calculated

Our web collection on statistics for biologists contains articles on many of the points above.

Software and code

Policy information about availability of computer code

Data collection	This study is based on the UK Biobank data release. No new data was collected for this study and hence no software was used for data collection.
Data analysis	The genotype data was handled with QCTOOL version 2.0.1 (https://www.well.ox.ac.uk/~gav/qctool_v2/) and PLINK version 1.90 (https://www.cog-genomics.org/plink/1.9/) and 2.00 (https://www.cog-genomics.org/plink/2.0/). IBD segments of siblings were inferred with snipar (https://github.com/AlexTISYoung/snipar/blob/ff48c642da1e45067afae1e21f5e5e450d4d4ef9/) and also with KING version 2.2.4 (https://www.kingrelatedness.com) for comparison. Statistical analysis were performed in python version 2.7.11 (https://www.python.org) and R version 3.4.3 (https://www.R-project.org/). LD score regression intercepts and estimates of heritability and genetic correlations were attained with LDSC version 1.0.1 (https://github.com/bulik/ldsc). GWAS summary statistics for the phenotypes shown in Table 1 were attained with BOLT-LMM version 2.3 (https://alkesgroup.broadinstitute.org/BOLT-LMM/). Python and R scripts for performing primary participation GWAS are available at https://github.com/stefaniabe/PrimaryParticipationGWAS.

For manuscripts utilizing custom algorithms or software that are central to the research but not yet described in published literature, software must be made available to editors and reviewers. We strongly encourage code deposition in a community repository (e.g. GitHub). See the Nature Portfolio guidelines for submitting code & software for further information.

Data

Policy information about availability of data

All manuscripts must include a data availability statement. This statement should provide the following information, where applicable:

- Accession codes, unique identifiers, or web links for publicly available datasets
- A description of any restrictions on data availability
- For clinical datasets or third party data, please ensure that the statement adheres to our policy

The primary participation GWAS summary statistics generated in this study have been deposited to GWAS catalog under the accession codes GCST90267220, GCST90267221, GCST90267222 and GCST90267223. Researchers can apply for access to individual-level UK Biobank data on their website (http://www.ukbiobank.ac.uk/register-apply/).

Field-specific reporting

Please select the one below that is the best fit for your research. If you are not sure, read the appropriate sections before making your selection.

☒ Life sciences ☐ Behavioural & social sciences ☐ Ecological, evolutionary & environmental sciences

For a reference copy of the document with all sections, see nature.com/documents/nr-reporting-summary-flat.pdf

Life sciences study design

All studies must disclose on these points even when the disclosure is negative.

Sample size	This study is based on all 313,860 genotyped individuals in the UK Biobank that fulfilled our relatedness, ancestry and quality control criteria. The primary participation GWASs were performed with 16,668 sibling pairs and 4,427 parent-offspring pairs. PGS analysis were performed with a non-overlapping group of 272,409 individuals.
Data exclusions	The sample was restricted to first-degree relative pairs with white British ancestry (the primary participation GWASs) and white British individuals with no relatives of 3rd degree or closer within the UK Biobank (the PGS analysis). The parent-offspring pairs and sibling pairs were chosen so that the two groups woulds not overlap. For sib-ships with more than two siblings, we chose the two first participating siblings. We excluded all individuals who had withdrawn consent, had a duplicate/twin in sample, had an excess of third degree relatives, were a heterozygosity or missingness outlier, had missing rate above 2%, were not included in the kinship inference of UK Biobank, were not included in the phasing input of UK Biobank, showed potential sex chromosome aneuploidy, demonstrated potential sex mismatch.
Replication	For each SNP, association with primary participation was tested in three non-overlapping groups (parent-offspring pairs, IBD1 sibling pairs and IBD2/IBD0 siblings pairs) resulting in three independent test-statistics (TNTC, WSPC and BSPC).
Randomization	The methodology introduced in this paper relies on the Mendelian model of inheritance, i.e. one of the two alleles in a parent is randomly transmitted to the offspring with equal probability.
Blinding	This is not relevant to our study as we did not compare different experimental groups.

Reporting for specific materials, systems and methods

We require information from authors about some types of materials, experimental systems and methods used in many studies. Here, indicate whether each material, system or method listed is relevant to your study. If you are not sure if a list item applies to your research, read the appropriate section before selecting a response.

Materials & experimental systems

n/a	Involved in the study	
☒	☐	Antibodies
☒	☐	Eukaryotic cell lines
☒	☐	Palaeontology and archaeology
☒	☐	Animals and other organisms
☐	☒	Human research participants
☒	☐	Clinical data
☒	☐	Dual use research of concern

Methods

n/a	Involved in the study	
☒	☐	ChIP-seq
☒	☐	Flow cytometry
☒	☐	MRI-based neuroimaging

Human research participants

Policy information about studies involving human research participants

Population characteristics	The UK Biobank is a prospective cohort that includes approximately 500 thousand genotyped and phenotyped individuals (45.6% male), aged 40-69 years old at recruitment, from across the United Kingdom.
Recruitment	Invitations to participate were sent to 9,238,453 individuals who were aged between 40 and 69 years and lived within 25-mile radius of any of the 22 UK Biobank assessment centres. Of those, 5.45% participated and went through baseline assessments that took place from 2006 to 2010. This has been described in detail by Fry et al. (2017) (https://doi.org/10.1093/aje/kwx246).
Ethics oversight	UK Biobank has approval from the North West Multi-centre Research Ethics Committee (MREC) as a Research Tissue Bank (RTB) approval. Research Ethics Committee reference 21/NW/0157.

Note that full information on the approval of the study protocol must also be provided in the manuscript.
